# iTRAQ-Based Quantitative Proteomics Unveils Protein Dynamics in the Root of *Solanum melongena* L. under Waterlogging Stress Conditions

**DOI:** 10.3390/life13061399

**Published:** 2023-06-15

**Authors:** Xu Yang, Zheng Jiang, Jie He, Lei Shen

**Affiliations:** College of Horticulture and Landscape Architecture, Yangzhou University, Yangzhou 225009, China; yangxu@yzu.edu.cn (X.Y.); mz120211371@stu.yzu.edu.cn (Z.J.);

**Keywords:** *Solanum melongena* L., proteome, iTRAQ, waterlogging, anaerobic metabolism

## Abstract

Waterlogging poses significant abiotic stress that endangers the survival of plants, including crops. In response, plants dramatically change their physiology to enhance their tolerance to waterlogging, such as proteome reconfiguration. Here, we utilized isobaric tags for the relative and absolute quantitation (iTRAQ)-based protein labeling technique to examine the proteomic changes induced by waterlogging in the roots of *Solanum melongena* L., a solanaceous plant. The plants were subjected to 6, 12, and 24 h of waterlogging stress at the flowering stage. Of the 4074 identified proteins, compared to the control, the abundance of the proteins increased and decreased in 165 and 78 proteins, respectively, in 6 h of treatments; 219 and 89 proteins, respectively, in 12 h of treatments; and 126 and 127 proteins, respectively, in 24 h of treatments. The majority of these differentially regulated proteins participated in processes such as energy metabolism, amino acid biosynthesis, signal transduction, and nitrogen metabolism. Fructose–bisphosphate aldolase and three alcohol dehydrogenase genes, in particular, were up- or down-regulated in waterlogging-treated *Solanum melongena* roots, suggesting that some proteins related to anaerobic metabolism (glycolysis and fermentation) may play vital roles in protecting its roots from waterlogging stress to enable long-term survival. Overall, this research not only offers a comprehensive dataset of protein alterations in waterlogged *Solanum melongena* roots but also insights into the mechanisms by which solanaceous plants adapt to waterlogging stress.

## 1. Introduction

Waterlogging, a condition that arises from poor drainage, partial or complete flooding, and extended periods of rainfall has been recognized to have negative impacts on the growth and yield of numerous vegetable crops [1,2]. This issue is becoming more and more prevalent as there is an increase in the number of plants exposed to flooding, largely due to erratic and unpredictable rainfall patterns globally. Excessive water accumulation in the soil can lead to hypoxic (low-oxygen) and anoxic (absence of oxygen) conditions within a few hours [3]. The hypoxic conditions hinder root respiration, which is vital for nutrient uptake and overall plant health and thus leads to reduced plant growth, epinasty (downward bending of leaves and stems), leaf chlorosis (yellowing), necrosis (cell death), and a decreased fruit yield [4]. In response to hypoxic conditions, plants employ various strategies to adapt and survive. One such strategy involves activating anaerobic pathways, with glycolysis being the primary pathway. Additionally, plants undergo morphological adaptations, including the formation of aerenchyma and adventitious roots [5]. Excess soil water not only impacts root respiration but also affects various physiological processes that are crucial for plant growth and yield. These processes include water relations (balance between water uptake and loss), the photosynthetic rate (PN) [6], stomatal opening (regulation of gas exchange) [7], assimilating translocation (movement of nutrients within the plant) [8], and nutrient uptake [9,10]. Furthermore, plants exposed to prolonged low-oxygen stress experience an increased accumulation of reactive oxygen species (ROS). While ROS plays an important role in normal plant physiology, excessive quantities can result in oxidative damage in plant cells, causing cellular dysfunction and potentially leading to cell death [11]. Therefore, plants have evolved regulatory mechanisms to withstand waterlogging stress. CmERF5 activates the ethylene-responsive VII transcription factor *CmRAP2.3* to enhance the tolerance of *Chrysanthemum morifolium* to waterlogging stress via the ROS pathway [12]. Overexpression of the sunflower transcription factor *HaHB11* enhanced the tolerance of maize to waterlogging and defoliation stress [13]. Overexpression of pyruvate decarboxylase 1 (*AdPDC1*) from *Actinidia deliciosa* enhanced the tolerance of Arabidopsis thaliana to waterlogging stress [14]. The waterlogging-responsive ERF transcription factor MaRAP2-4 from Mentha arvensis enhanced the tolerance of Arabidopsis thaliana to waterlogging stress via regulating the expression of the bidirectional sugar transporter AtSWEET10 [15]. These reports indicate that different regulatory mechanisms, such as ROS metabolism and glycometabolism, were activated in the plant response to waterlogging stress. However, these regulatory mechanisms in the plant response to waterlogging stress are largely unclear.

Proteomic methodologies offer a robust means for examining changes in plant proteomes as they respond to abiotic stress [16], as well as investigating various dimensions of plant physiology [17]. Comparative proteomics has been reported as an effective method for systematically exploring protein modifications in relation to a diverse array of abiotic stressors, such as salt stress [18], water stress [19,20,21,22], and temperature variations [23,24,25]. Among the proteomic techniques, isobaric tags for the relative and absolute quantitation (iTRAQ)-based proteomic method is particularly popular for conducting comparative analyses of multiple samples [26,27]. This approach is advantageous because it enables simultaneous peptide identification as well as quantification. This is achieved by labeling the peptides with isobaric tags and then analyzing them using tandem mass spectrometry (MS/MS) to measure the peak intensities of reporter ions. Consequently, iTRAQ allows researchers to draw meaningful comparisons and gain valuable insights into the proteomic changes under various abiotic stress conditions [28,29,30].

*Solanum melongena* (Solanaceae), commonly known as the eggplant, is a commercially significant and diversely cultivated vegetable throughout China [31,32]. Although these plants can flourish and grow in a variety of climates, they are particularly susceptible to excessive soil moisture. In southern China, *Solanum melongena* production faces significant challenges during the rainy season (May to July), when heavy and persistent rainfall is common. The excessive rainfall, coupled with inadequate drainage systems, results in waterlogging, which can, in turn, result in a reduction in oxygen levels in the soil, making it difficult for the plants to survive and potentially causing their sudden death. Given the importance of *Solanum melongena* as a vegetable crop, it is crucial to develop strategies to enhance its tolerance to waterlogging stress. Here, we waterlogged *Solanum melongena* plants for 6, 12, and 24 h, and the roots were used for an iTRAQ-based proteomic analysis. After exposing the plants to waterlogging stress, we collected root samples and analyzed them using iTRAQ-based proteomic techniques. This comprehensive methodology allowed us to identify specific proteins and molecular pathways that are potentially associated with the plant’s response to stress by waterlogging stimuli. By better understanding these proteomic-level physiological adaptations in *Solanum melongena* roots, researchers and agricultural experts can devise targeted interventions and breeding strategies to improve the waterlogging tolerance of this economically important crop.

## 2. Materials and Methods

### 2.1. Plant Sources, Cultivation Conditions, and Waterlogging Stress Interventions

Our study employed XN, a cultivated eggplant variety known for its high tolerance to waterlogging stress. The germination and seedling growth of XN were conducted in plugs containing a 1:1:1 mixture of vermiculite, loam, and perlite at 25 °C, under a 16 h light and 8 h dark cycle, with a relative humidity of 75–85%. Upon the development of four true leaves, the seedlings were categorized into four groups: Group 1 (acted as the control), Group 2 (plants underwent 6 h of waterlogging), Group 3 (plants experienced 12 h of waterlogging), and Group 4 (plants were subjected to 24 h of waterlogging). The latter three groups were submerged in a tank with the water level maintained at 1.0 cm in the plugs above the soil surface. For physiological and proteomic analyses, roots from both waterlogged and control condition plants were collected. The roots of 10 seedlings from the same replicate were combined, instantly frozen in liquid nitrogen, and stored at −80 °C for subsequent use.

### 2.2. Protein Isolation

The roots of *Solanum melongena* were finely ground in liquid nitrogen utilizing a pestle and mortar. The resulting powder was shifted to a 50 mL tube, and 25 mL of acetone/TCA (9:1) along with 65 mM DTT were added. The mixture was mixed and stored at −20 °C for 1 h. Following centrifugation at 10,000× *g* for 45 min, the supernatant was carefully removed. The residues were then suspended in STD buffer (1 mM DTT, 4% SDS, 150 mM Tris-HCl pH 8.0) in a volume of 10:1. The samples underwent ultrasonic disruption (80 watts, 10 s per cycle, 10 cycles). After centrifugation, the supernatants were stored at −80 °C for future use.

### 2.3. Protein Quantification and Proteolysis

The Bradford method was employed to determine total protein concentrations [33]. For each sample, 200 µg of protein was mixed with 200 µL of UA buffer (150 mM Tris-HCl pH 8.0, 8 M Urea) and transferred into a 10 kDa ultrafiltration centrifuge tube. The tube was centrifuged at 14,000× *g* for 15 min to remove impurities and concentrate the protein solution. Subsequently, 100 μL indoleacetic acid (IAA) (50 mM IAA in UA) was added to the concentrate, and the tube was centrifuged once again to promote the reduction and alkylation of proteins. Afterwards, the concentrate was diluted using 100 µL UA buffer and concentrated with another round of centrifugation. This dilution and concentration process was repeated twice to ensure the removal of residual contaminants. Next, 40 μL of trypsin buffer (2 μg trypsin in 4 μL dissolution buffer) was added to the proteins, which were then incubated at 37 °C overnight to enable efficient protein digestion into peptides. An equal volume of 0.1% formic acid (FA) was added to the mixture to halt enzymatic activity. The digested peptides were then purified using a Strata-XC18 column, washed with 5% acetonitrile (ACN) and 0.1% FA twice, and eluted with 1 mL 80% ACN and 0.1% FA. The eluted peptides were dried in a vacuum concentrator and redissolved in 500 μL of 0.5 M tetraethylammonium bromide (TEAB), which served as the buffer for the subsequent peptide labeling step.

### 2.4. iTRAQ Tagging and Separation

Samples were tagged using the iTRAQ Reagent-8 plex Multiplex Kit (AB Sciex, Cheshire, UK) as per the manufacturer’s guidelines. After labeling and quenching, the labeled samples were combined, lyophilized to remove solvents, and then reconstituted in 4 mL of 25% (*v*/*v*) ACN + 25 mM NaH_2_PO_4_ (pH 3.0) to prepare them for fractionation. Strong cation exchange (SCX) chromatography was conducted on a polysulfoethyl column (5 µm, 200 Å, 4.6 × 100 mm) (PolyLCInc, Columbia, MD, USA) using an AKTA Purifier 100 system (GE Healthcare, Danderyd, Sweden) with gradient elution to separate the peptides into eight distinct fractions based on their charge properties.

### 2.5. Capillary Liquid Chromatography–Tandem Mass Spectrometry (LC-MS/MS)

For capillary LC-MS/MS analysis of the collected peptide fractions, the Thermo Finnigan Q-Exactive Easy nLC-MS/MS system was utilized. Each peptide fraction was chromatographed using a carefully designed 60 min gradient elution, progressing from 0 to 35% (mobile phase A: 0.1% [*v*/*v*] FA in water; B: 0.1% [*v*/*v*] FA in 84% [*v*/*v*] ACN), ensuring optimal separation of peptides. The samples were first injected onto a Thermo Scientific EASY C18 trap column (2 cm × 100 μm, 5 μm), which served to desalt and concentrate the peptides before analysis. Following this, the peptides were subjected to separation using an analytical Thermo Scientific EASY C18 column (75 μm × 100 mm, 3 μm), maintaining a flow rate of 300 nL/min to achieve high-resolution separation. The precursor ion scan spectra were collected within a range of 300–1800 *m*/*z*, with MS1 acquisition settings configured as follows: spectrum resolution of 70,000 at *m*/*z* 200; AGC target: 3 × 10^6^; maximum IT: 10 ms; number of scan ranges: 1; and dynamic exclusion: 40.0 s. After each full MS scan, the instrument collected 10 data-dependent MS2 spectra to provide in-depth information about the peptide fragments. The MS2 acquisition settings were optimized for detailed analysis, including activation type; isolation window: 2 *m*/*z*; HCD; spectrum resolution: 17,500 at *m*/*z* 200; maximum IT: 60 ms; microscans: 1; underfill ratio: 0.1%; and normalized collision energy: 30 eV.

### 2.6. Statistical Analysis

The raw MS/MS data files produced through the capillary LC-MS/MS method were processed and examined using the advanced Mascot 2.2 and Proteome Discoverer 2.5 (Thermo Scientific, Waltham, MA, USA) software tools. To identify proteins, the acquired MS/MS spectra were subjected to a search against the comprehensive Eggplant Genome Database (http://eggplant-hq.cn/Eggplant/home/index (16 April 2023)) [32], with parameters set as biological modifications of ID focus, trypsin digestion, the Quantitate. Bias and background correction were checked for protein quantification and normalization. Generation of peak lists was performed with the Proteome Discoverer 2.5 software (Thermo Fisher Scientific, Waltham, MA, USA) [34]. Protein quantification and identification were carried out utilizing the Mascot software (V2.2, Matrix Science Inc., Boston, MA, USA). For the iTRAQ labeling quantification analysis, only unique peptides exhibiting a confidence level higher than 95% were included, ensuring the reliability of the results. Proteins with an unused value surpassing 1.2 were considered worthy of further investigation. To accurately evaluate the differences between the samples being compared, the fold change was calculated by considering the median abundance of replicate samples. Additionally, the *p*-values were determined using a rigorous Student’s *t*-test, which helped minimize false positives in the analysis. Proteins that exhibited a fold change greater than 1.5 and a *p*-value of less than 0.05 were considered to be a significantly different protein abundance. The mass spectrometry proteomics data have been deposited to the ProteomeXchange Consortium via the PRIDE partner repository with the dataset identifier PXD041747 [35,36].

### 2.7. Bioinformatics and Annotations

Gene Ontology (GO) terms (http://geneontology.org/ (16 April 2023)) are a set of standardized vocabularies that categorize gene products according to their related molecular functions, cellular components, and biological processes. These terms provide a unified language for describing and comparing genes and their products across different species. In this study, the identified protein sequences were mapped to their corresponding GO terms to gain a deeper understanding of their biological and functional properties [37]. Initially, a homology search was conducted for all the identified protein sequences using a local NCBI BLASTp program against the NCBI non-redundant (nr) database. The e-value threshold was set to less than 1 × 10^−5^. For each query sequence, the best match was selected for further GO term matching. The GO term matching was performed with blast2go and go2protein [https://www.blast2go.com/ (16 April 2023)] website [38]. To identify candidate biomarkers, we employed a hypergeometric test to perform GO and KEGG pathway enrichment [39].

## 3. Results

### 3.1. Stress Symptoms in Eggplant Seedlings during Waterlogging

**Figure 1:**
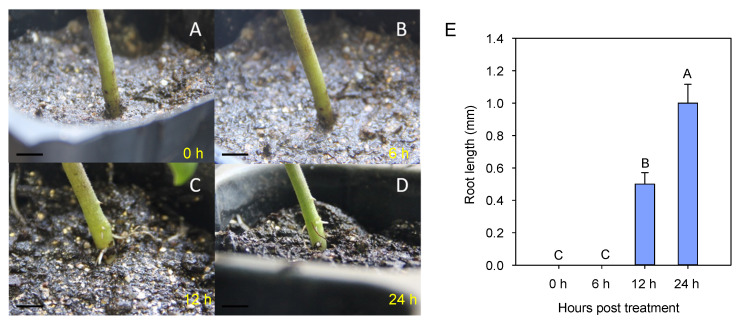
Different responses of eggplant seedlings to waterlogging treatments. (**A**) No waterlogging; (**B**) 6 h, (**C**) 12 h, and (**D**) 24 h waterlogging treatments. Bar = 10 mm. (**E**) The new root length of eggplant with the waterlogging stress treatment at the time points of 0, 6, 12, and 24 h post treatment. Different capital letters between samples represent significant differences—one-way ANOVA test (*p* < 0.01).

Identification of waterlogging tolerance conducted with morphological indexes was intuitive, simple, and fast. In this study, waterlogging conditions were applied to eggplant seedlings with four main leaves for 24 h. As shown in Figure 1, symptom changes were displayed at four different time points (0, 6, 12, and 24 h). Notably, a significant symptom was that some litter white adventitious roots on the surface of the eggplant stem above substrates had taken shape after a 12 h waterlogging treatment (Figure 1C), but no obvious changes could be found after 6 h (Figure 1B). The average length of the adventitious roots measured at 6, 12 (Figure 1B,C), and 24 h (Figure 1D) was 0, 0.5, and 1.0 mm, respectively. Interestingly, the number of roots did not change between the 12 and 24 h treatments. This consistency might result from the short waterlogging times used in this experiment. In addition, we selected some genes induced by waterlogging stress from the data of protein quantification and the differential analysis of the eggplant with a real-time quantitative PCR assay at the time points of 0, 6, 12, and 24 h post waterlogging treatment (Supplementary Table S1), and the results show that the relative transcript expression levels of selected genes including galactoside 2-alpha-L-fucosyltransferase (*SmFUT1*, Smechr0101652.1) [40], peptidyl–prolyl *cis*-trans isomerase FKBP42 (*SmFKBP42*, Smechr0102011.1) [41,42], novel plant SNARE 13 (*SmSNARE13*, Smechr0502662.1) [43,44], the NAD-dependent protein deacylase SRT2 (*SmSRT2*, Smechr1102733.1), and the ultraviolet-B receptor UVR8 (*SmUVR8*, Smechr0300744.1) were significantly up-regulated under the condition of waterlogging stress (Figure S1, Table S4). Our results reveal that the effects of waterlogging stress on eggplant seedlings appeared at an early stage.

### 3.2. Protein Response to Waterlogging Stress in the Root of Solanum Melongena Identified with GO Analysis

To establish the changes in proteins in the roots of *Solanum melongena*, the gel-free iTRAQ analysis was employed. Expression profiles of the proteins of plants under waterlogging stress conditions for 0, 6, 12, and 24 h were assessed in two independent iTRAQ experiments. The ProteinPilot cut-off score for identifying proteins was set at 1.3, which corresponded to a confidence level of 95%. The data collected from the roots of *Solanum melongena* were assessed using Mascot software vs. 2.2 (Matrix Science, London, UK, which resulted in a total of 4074 proteins in these samples (Supplementary Table S1).

**Figure 2:**
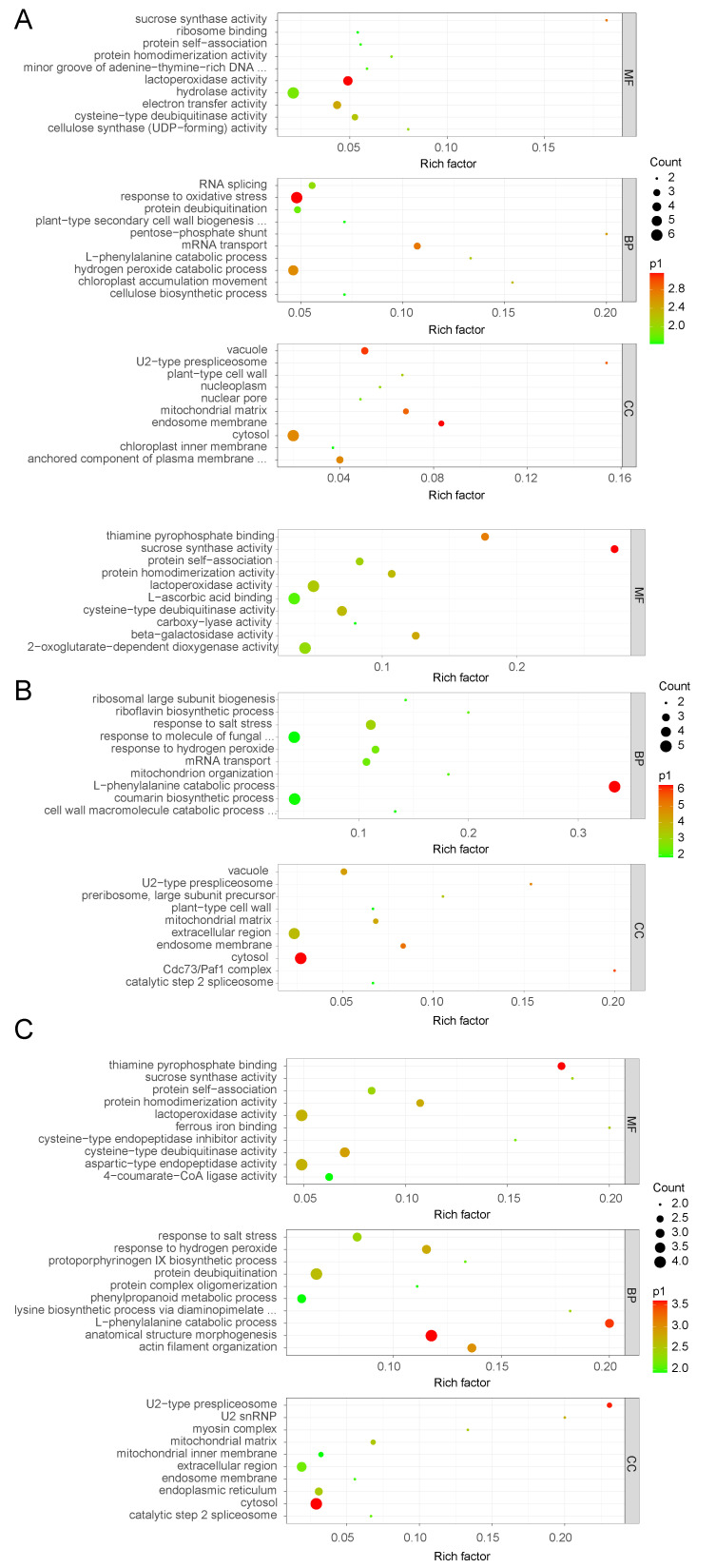
Categorization of different abundance proteins in 6 h/0 h (**A**), 12 h/0 h (**B**), and 24 h/0 h (**C**) groups based on GO terms for biological process, cellular component, and molecular function.

Compared with the control, we found that for Group 2, 165 proteins increased (Group 2: Group 1 ratio greater than 1.2, q-value less than 0.05) and 78 decreased (Group 2: Group 1 ratio less than 0.83, q-value less than 0.05). In addition, for Groups 3 and 4, 219 and 89 proteins exhibited increased levels, and 126 and 127 proteins exhibited decreased levels, respectively. Using the WEGO (Web Gene Ontology Annotation Plot) online website (https://wego.genomics.cn/) [45], we sorted all the discovered proteins into three major classes based on their GO annotations: molecular functions, cellular components, and biological processes. The GO analysis depicted that many different abundance proteins in Groups 2, 3, and 4 were engaged in diverse cellular, single-organism, metabolic, regulatory, and stimulatory processes (Figure 2). Consequently, waterlogging stress greatly remodels the proteome of *Solanum melongena* roots by influencing many dimensions of plant physiology. Most of the different abundance proteins were predicted to be associated with the cell, organelle, membrane, and macromolecular complexes, all of which fall under the umbrella of cellular components and are, therefore, likely to play a critical function in regulating the waterlogging-induced signaling of the cellular metabolic pathways. Most different abundance proteins in the eggplant response to waterlogging stress were classified as having molecular functions associated with binding or catalysis.

**Figure 3:**
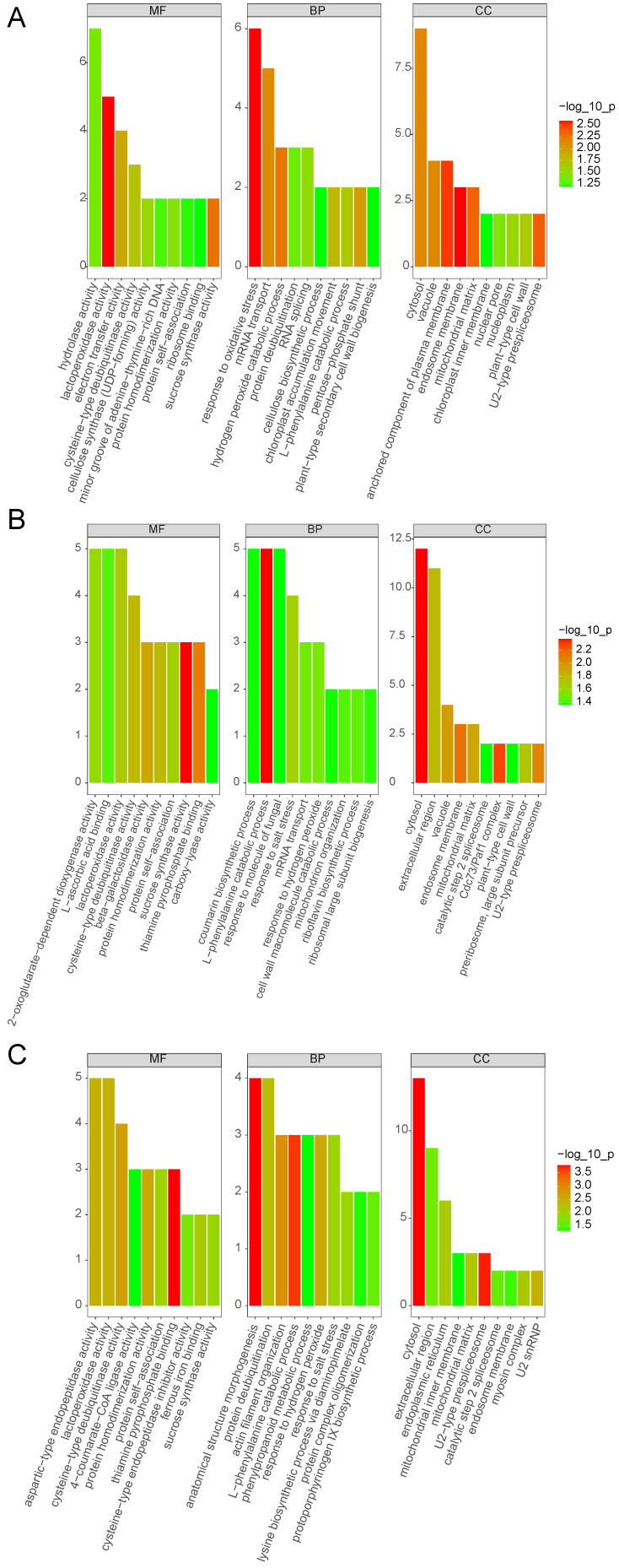
GO enrichment analysis for different abundance proteins in 6 h/0 h (**A**), 12 h/0 h (**B**), and 24 h/0 h (**C**) groups, considering cellular component, biological process, and molecular function using a hypergeometric statistical test.

The UniProt database (https://www.uniprot.org/ (accessed on 30 May 2023)) was used to retrieve the GO annotations of each important protein. Using a custom Perl script, we searched our in-house GO database to determine how many proteins were associated with each GO item [46]. For the enrichment analysis, we calculated the *p*-value of each GO item by running a hypergeometric test and then applying False Discovery Rate (FDR) multiple adjustments. Proteins engaged in biological processes were discovered at a higher rate than those involved in cell components or molecular function across all three treatment groups (Supplementary Table S2 and Figure 3).

### 3.3. Analysis of KEGG Pathway Enrichment of Eggplant Treated with Waterlogging Stress

**Figure 4:**
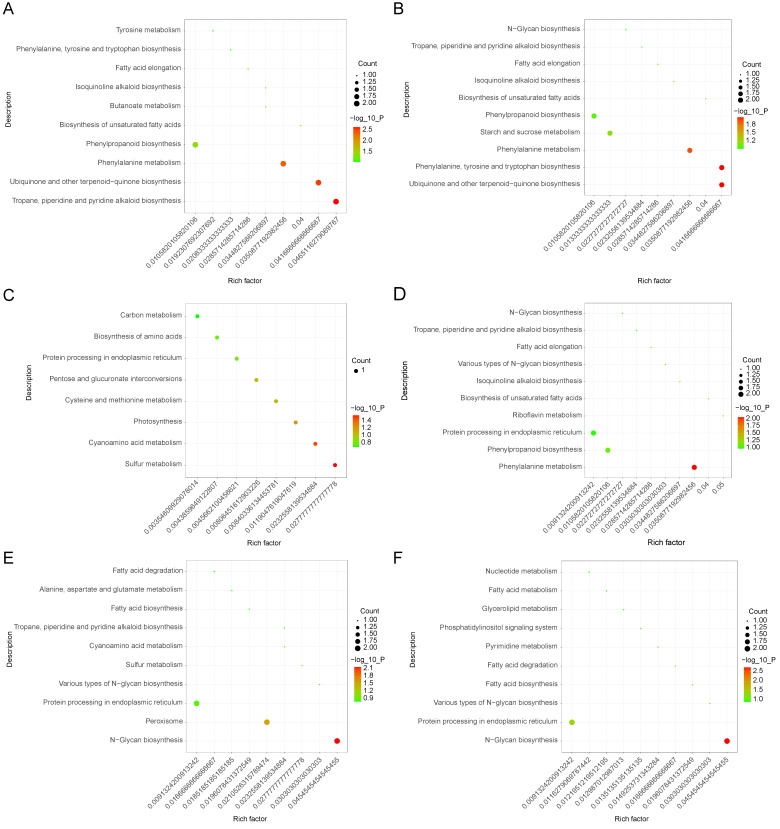
KEGG pathway analysis for different abundance proteins in 6 h/0 h (**A**), 12 h/0 h (**B**), 12 h/6 h (**C**), 24 h/0 h (**D**), 24 h/6 h (**E**), and 24 h/12 h (**F**) groups.

For an outline of the primary biochemical metabolic and signal transduction pathways that are affected by waterlogging stress in *Solanum melongena* roots, different abundance proteins were analyzed based on the Kyoto Encyclopedia of Genes and Genomes (KEGG; http://www.genome.jp (16 April 2023)), which according to the related biochemical pathways, provides an alternative functional annotation of the proteins (Supplementary Table S3 and Figure 4) [47]. Compared to the control group (0 h), the differentially abundant annotated proteins of the top 10 enrichment pathways in the 6 h/0 h group were related to tyrosine metabolism; phenylalanine, tyrosine, and tryptophan biosynthesis; fatty acid elongation; isoquinoline alkaloid biosynthesis; butanoate metabolism; the biosynthesis of unsaturated fatty acids; phenylpropanoid biosynthesis; phenylalanine metabolism; ubiquinone and other terpenoid−quinone biosynthesis; and tropane, piperidine, and pyridine alkaloid biosynthesis (Figure 4A). In the 12 h/0 h group, the different abundance proteins were associated with N−Glycan biosynthesis; tropane, piperidine, and pyridine alkaloid biosynthesis; fatty acid elongation; isoquinoline alkaloid biosynthesis; the biosynthesis of unsaturated fatty acids; phenylpropanoid biosynthesis; starch and sucrose metabolism; phenylalanine metabolism; phenylalanine, tyrosine, and tryptophan biosynthesis; and ubiquinone and other terpenoid−quinone biosynthesis (Figure 4B). In the 12 h/6 h group, differentially abundant proteins in the top eight enrichment pathways were related to carbon metabolism; amino acid biosynthesis; protein processing in the endoplasmic reticulum; pentose and glucuronate interconversions; cysteine and methionine metabolism; photosynthesis; cyanoamino acid metabolism; and sulfur metabolism (Figure 4C). The different abundance proteins in the 24 h/0 h group of the top 10 enrichment pathways were involved in N−Glycan biosynthesis; tropane, piperidine, and pyridine alkaloid biosynthesis; fatty acid elongation; various types of N−Glycan biosynthesis; isoquinoline alkaloid biosynthesis; the biosynthesis of unsaturated fatty acids; riboflavin metabolism; protein processing in the endoplasmic reticulum; phenylpropanoid biosynthesis; and phenylalanine metabolism (Figure 4D). Further comparison showed that the differentially abundant proteins in the 24 h/6 h group were related to fatty acid biosynthesis and degradation; alanine, aspartate, and glutamate metabolism; tropane, piperidine, and pyridine alkaloid biosynthesis; cyanoamino acid metabolism; sulfur metabolism; various types of N−Glycan biosynthesis; protein processing in the endoplasmic reticulum; the peroxisome; and N−Glycan biosynthesis (Figure 4E). In the 24 h/12 h group, the differentially abundant proteins in the top 10 enrichment pathways were related to nucleotide metabolism; the biosynthesis, metabolism, and degradation of fatty acids; glycerolipid metabolism; the phosphatidylinositol signaling system; pyrimidine metabolism; various types of N−Glycan biosynthesis; protein processing in the endoplasmic reticulum; and N−Glycan biosynthesis (Figure 4F). Our analysis revealed that different KEGG pathways were implicated in different waterlogging-treatment times.

### 3.4. Protein–Protein Interaction Analysis

**Figure 5:**
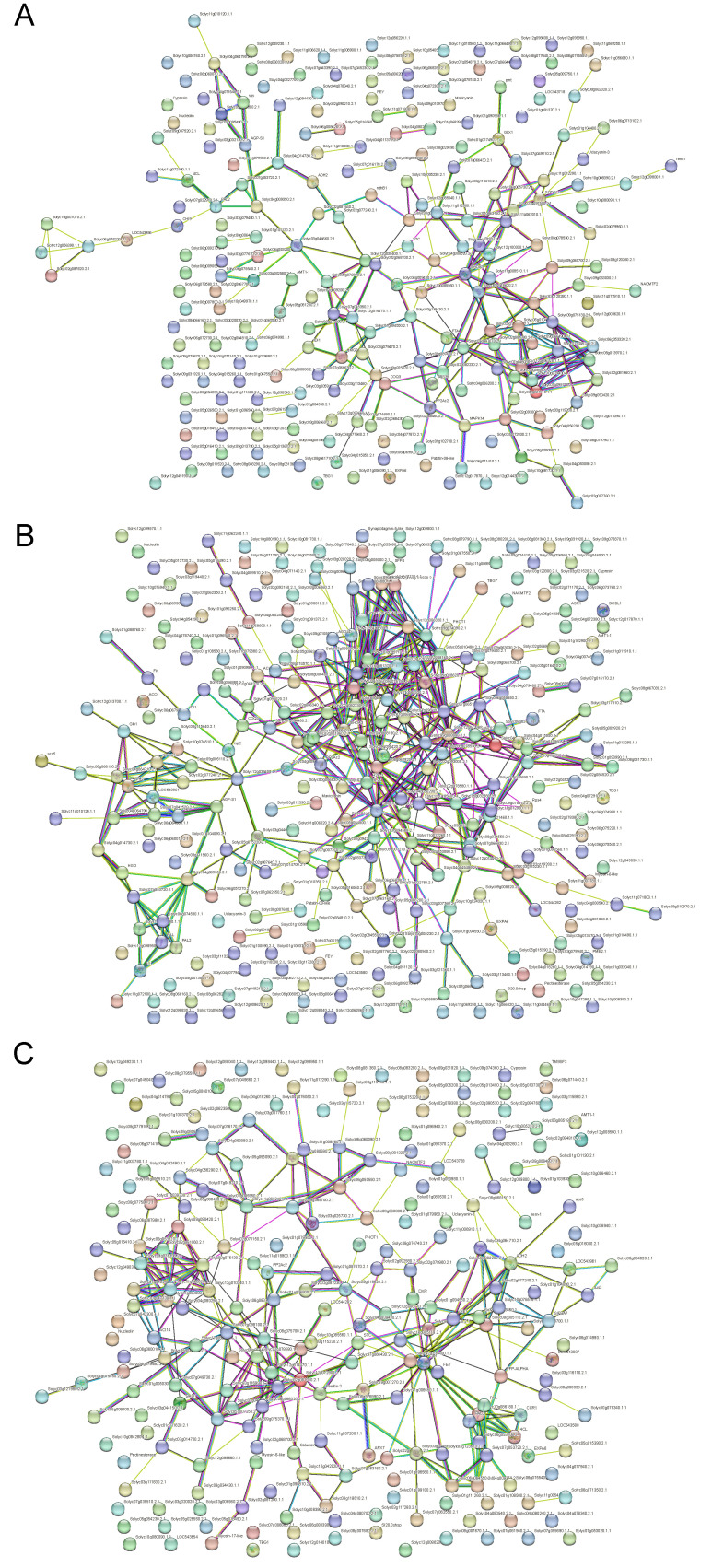
Protein–protein interaction network analyzed using STRING website: (**A**) network derived from significantly altered proteins in the 6 h/0 h sample group, (**B**) 12 h/0 h sample group, and (**C**) 24 h/0 h sample group. Various line colors indicate different types of association evidence: neighborhood evidence is shown with the green line, experimental evidence is shown with the purple line, fusion evidence is shown with the red line, database evidence is shown with the light blue line, co-occurrence evidence is shown with the blue line, co-expression evidence is shown with the black line, and text-mining evidence is shown with the yellow line.

STRING, a free tool available at http://string-db.org/ (16 April 2023), was used to examine the protein–protein interaction network. Using sequence alignment, the significantly different abundance proteins were mapped to the STRING database using the standalone BLAST program (version 2.2.27) (NCBI, Bethesda, MD, USA). A molecular network among these proteins was constructed according to the protein–protein interactions from STRING. The network was then visualized using Cytoscape (version 2.8.3) software. Figure 5 displays the important protein interactions between different abundance proteins. In Group 2, these protein interactions included FEY (Forever young oxidoreductase), Mavicyanin (Cupredoxin superfamily protein), AMT1-1 (Ammonium transporter 1 member 1), PP2Ac2 (Serine/threonine–protein phosphatase), AGP-S1 (Glucose-1-phosphate adenylyltransferase), sps (Sucrose phosphate synthase), MAPK14 (Mitogen-activated protein kinase 14), and ndhB1 (NAD(P)H-quinone oxidoreductase subunit) (Figure 5A). In Group 3, these protein interactions included TBG7 (Beta-galactosidase), SlFBA7 (fructose–bisphosphate aldolase), ribA (GTP cyclohydrolase), ispE (4-diphosphocytidyl-2-C-methyl-D-erythritol kinase), ACO1 (1-aminocyclopropane-1-carboxylate oxidase 1), PME2.1 (Pectinesterase 2.1), Glb1 (Non-symbiotic hemoglobin class 1), IDI1 (Plastid isopentenyl diphosphate isomerase), P18 (Deoxyuridine 5’-triphosphate nucleotidohydrolase), FTA (Protein farnesyltransferase), H2B-3 (Histone H2B.3), PHOT1 (Phototropin 1), and SFP4 (Sugar transporter) (Figure 5B). In Group 4, ADH2 was included in the following protein interactions: cat1 (Catalase isozyme 1), TBG1 (Beta-galactosidase), DAD1 (Dolichyl-diphosphooligosaccharide-protein glycosyltransferase subunit DAD1), and CCR1 (Cinnamoyl-CoA reductase) (Figure 5C). These proteins have critical roles in cellular signaling, energy and lipid metabolism, and immune system function. For example, by converting nicotinamide adenine dinucleotide (NAD+) to nicotinamide adenine dinucleotide (NADH), alcohol dehydrogenase enables the interconversion of alcohols to aldehydes or ketones. Under normal physiological conditions, young plants grown in agar show low-level, constitutive expression of ADH in their root systems [48]. Under the condition of oxygen deprivation, dehydration, and low temperatures, the protein abundance of ADH is significantly increased [49]. In addition, we provided a simplified interacting net of some critical proteins based on the protein–protein interaction analysis in the eggplant response to waterlogging stress at 6, 12, and 24 h post treatment comparing to 0 h (Figure S2).

### 3.5. Analysis of Waterlogging-Related Proteins

Compared with the control condition, the up-regulated and annotated proteins in Group 2 included fructose–bisphosphate aldolase, 14-3-3-like protein A, the T-complex protein 1 subunit, DnaJ protein, Serine/threonine–protein phosphatase, alcohol dehydrogenase, the eukaryotic translation initiation factor 3 subunit, and 70 kDa peptidyl–prolyl isomerase (Supplementary Table S1). The up-regulated and annotated proteins in Group 3 included not only those in Group 2 but also multi-protein bridging factor-like, *S*-adenosylmethionine synthase, Argonaute104, Triosephosphate isomerase, and 40S ribosomal protein, which was the 60S ribosome, actin, 60 kDa chaperonin, Serine/threonine–protein phosphatase, fructose–bisphosphate aldolase, Triosephosphate isomerase, and alcohol dehydrogenase in Group 4. Among these differentially abundant proteins, alcohol dehydrogenase and fructose–bisphosphate aldolase were disclosed in the three treated groups. Fructose–bisphosphate aldolase was the most up-regulated protein in Groups 2 (1.35-fold increase) and 3 (1.49-fold increase), suggesting that waterlogging stress may affect gene transcription via the modification of gluconeogenesis and the glycolysis pathway. However, three fructose–bisphosphate aldolases in Groups 2 and 3 were involved in the waterlogging stress, and one of them was down-regulated. Strikingly, three identified fructose–bisphosphate aldolases in Group 4 were all up-regulated, with a 1.8-fold increase. These analyses indicate that when adapting to waterlogging stress, some types of fructose–bisphosphate aldolase need to be reduced at some time point. Further comparisons among the waterlogging-treated groups indicate that the abundance of fructose–bisphosphate aldolase increased the longer the plants were waterlogged (Supplementary Table S1). Alcohol dehydrogenase was the most up-regulated protein in Group 4, with a 2.52-fold increase. Moreover, its increase in Groups 2 (1.23-fold increase) and 3 (1.49-fold increase) was observed. The results of the analysis on alcohol dehydrogenase suggest that it had a strong, positive response to waterlogging stress.

Regarding the significantly repressed proteins under waterlogging stress, they have a characteristic of diversity at different times. In Group 2, 10 annotated proteins were markedly down-regulated, namely heterogeneous nuclear ribonucleoprotein A2/B1-like, the elongation factor Tu, ATPase 3, histone deacetylase, nucleoside diphosphate kinase, pyruvate kinase, adenosine triphosphate (ATP) synthase, ATP-dependent zinc metalloprotease, peptidyl–prolyl cis-trans isomerase, and RuBisCO large subunit-binding protein. In Group 3, there were eight annotated proteins, namely heterogeneous nuclear ribonucleoprotein A2/B1-like, ATP synthase, aminomethyltransferase, histone deacetylase, nucleoside diphosphate kinase, serine hydroxymethyltransferase, 12-oxophytodienoate reductase, and Serine/threonine–protein phosphatase. In Group 4, there were six annotated proteins that were involved, namely heterogeneous nuclear ribonucleoprotein A2/B1-like, the elongation factor Tu, pyruvate kinase, aminomethyltransferase, nucleoside diphosphate kinase, and Serine/threonine–protein phosphatase. These down-regulated proteins were all negatively affected by waterlogging stress.

## 4. Discussion

When the amount of water in the soil exceeds its absorption capacity, a certain amount of water stays on the surface of the soil, which will eventually lead to the occurrence of waterlogging stress [50]. To better understand the physiological mechanisms that underlie plant stress responses, investigation is currently conducted on plant responses to abiotic stress stimuli at the proteome level [16]. The iTRAQ-based proteomic assessment was conducted to understand how the function of proteins changes in the roots of *Solanum melongena* under stress with waterlogging and to clarify the molecular mechanisms responsible for waterlogging tolerance. The iTRAQ-based quantitative method is a strong proteomic tool for the identification of different abundance proteins to understand the variations in the plant proteome in response to stress with abiotic stimuli [51]. Through iTRAQ, researchers are able to identify five specific physiological parameters, including the concentration of malondialdehyde (MDA), alcohol dehydrogenase activity, GSH, nicotinamide adenine dinucleotide, and adenosine 5’-triphosphate [52]. These changes are consistent with the proteomic changes observed in maize roots in response to waterlogging stress [52].

The iTRAQ-based quantitative approach led to the identification of 4235 waterlogging-responsive proteins. The GO assessment depicted that these revealed proteins were implicated in cellular components, biological processes, and molecular functions. The further gene enrichment analysis suggested that the majority of these proteins were engaged in biological processes, followed by molecular function and cellular components. In the plant, the different proteins were inter-coordinated to exhibit their biological behavior, and the analysis based on the pathway helped us understand its biological function. At the proteome level, waterlogging has been found to induce variations in the abundance of proteins linked to various processes, such as photosynthesis, energy metabolism, signal transduction, redox homeostasis, RNA processing, PCD, protein biosynthesis, disease resistance, defense, and stress mechanisms [53,54]. In this study, waterlogging stress led to variations in the protein abundance found in *Solanum melongena* roots, which were implicated in several of these processes.

Plants’ responses to stress are dynamic processes that vary with the nature and severity of the stress they experience [55]. Various stages related to plant stress responses, which were characterized by their unique proteome composition, have been distinguished. In our study, *Solanum melongena* roots, under waterlogging stress for 6, 12, and 24 h, respectively, showed dissimilar different abundance proteins, suggesting that the amount of time the roots were placed under stress affected their response. Increased concentration of so-called anaerobic proteins, such as alcohol dehydrogenase and fructose–bisphosphate aldolase, is associated with the promotion of anaerobic metabolism, especially fermentation and glycolysis, in waterlogged plant roots [56]. The enhancement of alcohol dehydrogenase and fructose–bisphosphate aldolase in the roots of *Solanum melongena* because of waterlogging stress was also observed in this study, suggesting their strong positive response to waterlogging stress. This result is similar to that of a report that showed the improvement of waterlogging tolerance in plants pretreated with ALA [57]. In contrast, Xu et al. found that the waterlogging tolerance index of *Brassica napus* L. was independent of alcohol dehydrogenase [58]. Therefore, our study may provide some new insight for understanding the response of *S melongena* to waterlogging stress. Interestingly, more than one fructose–bisphosphate aldolase identified in the roots of *Solanum melongena* were induced by waterlogging stress, but not all of them were up-regulated, implying that some types of fructose–bisphosphate aldolase need to be down-regulated at some point in order to activate adaptations to waterlogging stress. However, the increased degree of their abundance was not significant as the highest increase observed was 2.5-fold. This may be attributed to the short length of time the plants were placed under waterlogging stress in this study. Apart from alcohol dehydrogenase and fructose–bisphosphate aldolase, many other waterlogging-responsive proteins, such as T-complex protein 1, 14-3-3-like protein A, and Serine/threonine–protein phosphatase, were up-regulated. Energy metabolism plays an important role in the physiological regulation of plant adaptation to hypoxia [59]. In the case of plant consumption of a carbohydrate, the ATP produced by anaerobic respiration is far less than that produced by aerobic respiration [50].

During the waterlogging of plants, the production of ATP is limited because of anaerobic metabolism. In our study, the abundance of ATP synthase and pyruvate kinase under waterlogging stress was markedly down-regulated. One molecule of ATP is produced when phosphoenolpyruvate transfers a phosphate group to adenosine diphosphate via the latter’s catalytic activity [60]. An analysis of tolerant and susceptible sesame revealed that waterlogging stress response patterns were implicated in energy metabolism and genes in the pathway of benzene synthesis [61]. Similar reports were made in various other studies with a variety of plant species, including Arabidopsis (*Arabidopsis thaliana*) [62], barley (*Hordeum vulgare*) [63], soybeans (*Glycine max*) [64], and potatoes (*Solanum tuberosum*) [65] exposed to waterlogging conditions. Apart from energy-related proteins, proteins involved in RNA splicing (heterogeneous nuclear ribonucleoprotein A2/B1-like), DNA wrapping (histone deacetylase), protein synthesis (the elongation factor Tu), carbon fixation (RuBisCO large subunit-binding protein), and amino acid metabolism (aminomethyltransferase) were all involved, further confirming the results of previous studies [54,66,67,68,69]. According to the KEGG analysis, we found that waterlogging stress can induce the accumulation of protein abundance in phenylpropanoid metabolism and the tropane, piperidine, and pyridine alkaloid biosynthesis pathway. Plant roots produce a lot of ROS due to a lack of oxygen in the condition of waterlogging stress [70]. Sucrose acted as a signaling molecule, and can directly mediate the phenylpropanoid metabolism (Smechr0500713.1, Smechr0402501.1), and tropane, piperidine, and pyridine alkaloid metabolism (Smechr0702092.1, Smechr0601455.1), which play an important role in maintaining osmotic balance and removing ROS [71]. Previous studies showed that the tropane, piperidine, and pyridine alkaloid biosynthesis pathway was triggered by drought and saline–alkali stress [71,72]. However, we did not find any reports about the tropane, piperidine, and pyridine alkaloid biosynthesis pathway’s involvement in the plant response to waterlogging stress. In addition, we also found that the peroxisome pathway (Smechr0401398.1, Smechr0102162.1) was enriched in the 24 h/6 h group (Figure 4E), suggesting that waterlogging stress activated the gene expression of the peroxisome pathway to eliminate excessive ROS to enhance the tolerance of the eggplant to waterlogging stress.

This study used the iTRAQ technology to give a comprehensive analysis of protein dysregulation in the roots of *Solanum melongena* during exposure to waterlogging stress and subsequent recovery. A total of 4235 proteins that had a different abundance were identified under waterlogging conditions. Based on the findings from this research, we propose that certain proteins related to anaerobic metabolism (glycolysis and fermentation) may play vital roles in protecting the roots of *Solanum melongena* from waterlogging stress to enable long-term survival. Studies on the mechanism of *Solanum melongena* under waterlogging resistance could not only provide guidance for the breeding of waterlogging-tolerant varieties but also be applied to the genetic improvement of other crops using the APX of *Solanum melongena* to modify the rice seed germination and early seedling resistance to waterlogging [73]. Based on these findings, the understanding of the mechanisms underlying *Solanum melongena*’s response to waterlogging stress has been enhanced, and research strategies can be further optimized. Subsequent research should integrate transcriptomic, proteomic, and metabolomic methodologies to reveal the extensive molecular interactions occurring in the roots of *Solanum melongena* as a response to waterlogging stress.

## Data Availability

Data are openly available in a public repository, please visit ProteomeXchange Consortium database (http://www.proteomexchange.org/) via the PRIDE partner repository with the dataset identifier PXD041747.

## Supplementary Materials

The following supporting information can be downloaded at: https://www.mdpi.com/article/10.3390/life13061399/s1, Figure S1: Analysis of transcript expression levels of waterlogging stress defense-related genes including galactoside 2-alpha-L-fucosyltransferase (*SmFUT1*, Smechr0101652.1), pep-tidyl-prolyl cis-trans isomerase FKBP42 (*SmFKBP42*, Smechr0102011.1), novel plant SNARE 13 (*SmSNARE13*, Smechr0502662.1), NAD-dependent protein deacylase SRT2 (*SmSRT2*, Smechr1102733.1), and ultraviolet-B receptor UVR8 (*SmUVR8*, Smechr0300744.1) in eggplant root treated with waterlogging stress using RT-qPCR assay at 24 h post treatment. Different capital letters between samples represent significant differences—one-way ANOVA test (*p* < 0.01); Figure S2: The simplified interacting net of some critical proteins based on the protein–protein interaction analysis in eggplant response to waterlogging stress at 6 (A), 12 (B), and 24 h (C) post treatment comparing to 0 h. Table S1: protein quantification and different abundance analyses of eggplant root at 0, 6, 12, and 24 h post waterlogging stress treatment; Table S2: The GO analysis of eggplant root treated with waterlogging stress at 0, 6, 12, and 24 h; Table S3: KEGG pathway enrichment analysis of eggplant root treated with waterlogging stress at 0, 6, 12, and 24 h; Table S4: Sequences of primer pairs were used in this study.
