# Supplementary material for: iTRAQ-Based Quantitative Proteomics Unveils Protein Dynamics in the Root of Solanum melongena L. under Waterlogging Stress Conditions
Source: Life (Basel). 2023 Jun 15;13(6):1399. doi: 10.3390/life13061399 (PMC10302559; doi:10.3390/life13061399)
Supplement: Supplementary file 1 [file life-13-01399-s001.zip › Table S4.pdf]

Table S4. Sequences of primer pairs were used in this study.

Primer name	Forward (5' to 3')	Reserves (5' to 3')	
SmFUT1	AGGCTTTGCATTCATTTTGG	CGATAGGCCTGTTCAAGCTC	RT-qPCR
SmFKBP42	GATTGGGGCAGCAGATAGAA	TCCCGGAACTTACCAAACAG	RT-qPCR
SmSNARE13	AAGCCGGGACAAGATTTCAT	TCCACGAATAGCATTCCTCTG	RT-qPCR
SmSRT2	CCGGCTTATCAAAGCTGCTA	CTCTCCAACTCGAGCACTGA	RT-qPCR
SmUVR8	CCCATCACCAGCTGAAAAAT	TTTCCGGCACATTTACATCA	RT-qPCR
SmActin	CACTTAGCACCTTCCAGCAGATGT	GTACAACAGCAGACCTGAGTTCACT	RT-qPCR
