# Supplementary figures and images for: iTRAQ-Based Quantitative Proteomics Unveils Protein Dynamics in the Root of Solanum melongena L. under Waterlogging Stress Conditions
Source: Life (Basel). 2023 Jun 15;13(6):1399. doi: 10.3390/life13061399 (PMC10302559; doi:10.3390/life13061399)

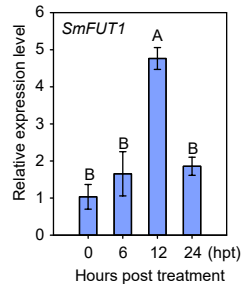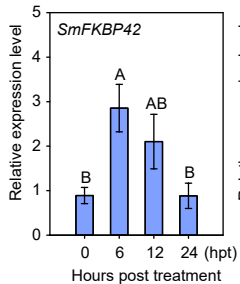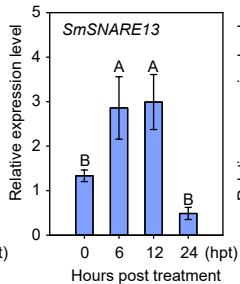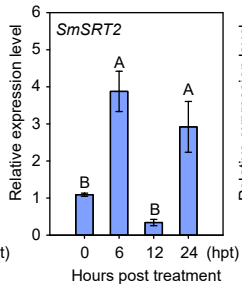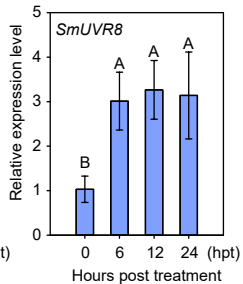

Supplement: Supplementary file 1 [file life-13-01399-s001.zip › Figure S1.pdf]

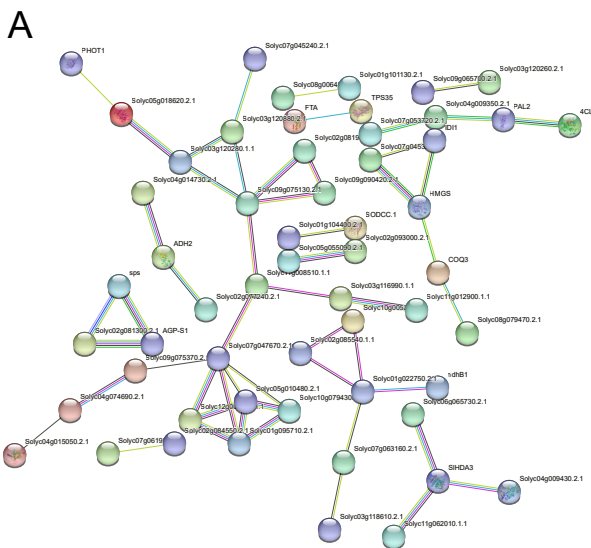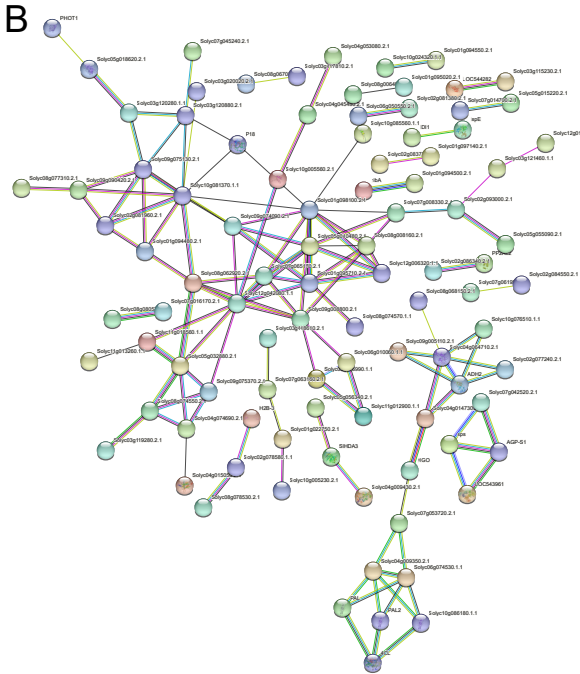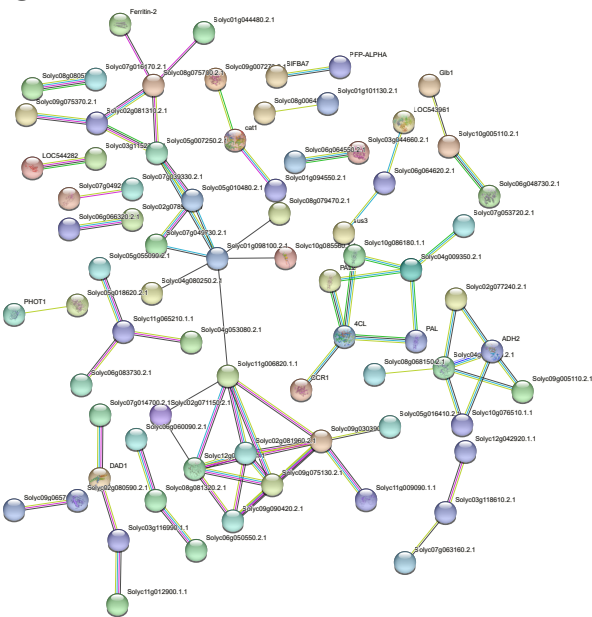

Supplement: Supplementary file 1 [file life-13-01399-s001.zip › Figure S2.pdf]
